# An In-Vitro Evaluation of the Characteristics of Zein-Based Films for the Release of Lactobionic Acid and the Effects of Oleic Acid

**DOI:** 10.3390/polym13111826

**Published:** 2021-05-31

**Authors:** Alessandro Coroli, Roberta Romano, Andrea Saccani, Noura Raddadi, Elisa Mele, Leno Mascia

**Affiliations:** 1 Department of Civil, Chemical, Environmental and Materials Engineering, University of Bologna, 40131 Bologna, Italy; alessandrocoroli@hotmail.it (A.C.); roberta.romano10@unibo.it (R.R.); andrea.saccani@unibo.it (A.S.); noura.raddadi@unibo.it (N.R.); 2 Materials Department, Loughborough University, Loughborough LE11 3TU, UK

**Keywords:** lactobionic acid, oleic acid, zein, drug delivery, antioxidant activity, antimicrobial activity

## Abstract

Lactobionic acid (LBA) is widely used in different industrial sectors owing to its biocompatibility characteristics as well as antioxidant and antimicrobial properties. In this study, mixtures of the protein zein with LBA and with the addition of oleic acid (OA) as a ternary system were investigated as drug delivery films for the release of LBA. The chosen combinations exploit the vast difference in water solubility between LBA and the other two components (zein and OA). DSC thermograms and dynamic mechanical spectra, alongside electron microscopy images, were used to describe the microstructural features of the films and were found to provide insights for the release of LBA from the two examined zein-based films immersed in an aqueous physiological solution. For both film systems, a burst release behavior was observed, followed by a rapid and total extraction of LBA. The required immersion time for the total extraction of LBA was greatly reduced when oleic acid was added to the precursor solution mixture for producing the films. The LBA released from the zein-based films was found to exhibit both the expected antioxidant properties as well as exerting bacteriostatic effects towards *Escherichia coli* and *Staphylococcus epidermidis*.

## 1. Introduction

Lactobionic acid (LBA) is obtained by the oxidation of lactose (the main carbohydrate of milk), and it can be produced from pure lactose or by-products of the dairy industry, such as cheese whey [1,2,3,4,5]. It is composed of a galactose moiety linked to a gluconic acid molecule via an ether-like linkage. The high content and proximity of hydroxyl groups of the LBA molecule are largely responsible for the high solubility of LBA in water (free water); while the strong intermolecular attractions with COOH groups through H-bonds with water molecules (bond water) are likely to determine moisture retention characteristics up to a temperature higher than the boiling point of water and close to the glass transition temperature (*T*_g_) of LBA [6].

Different industrial sectors, from the pharmaceutical to food industry, benefit from the properties of LBA, including metal-chelating and antioxidant activities, biocompatibility, and biodegradability [7,8]. LBA is the main ingredient of cold-storage solutions for organ transplantation because it reduces oxidative damages, catalyzed by metal ions, to tissues/organs. These potent antioxidant properties are of interest in skincare, where LBA is used to facilitate wound healing, inhibit the production of both hydroxyl radicals and matrix metalloproteinases that contribute to skin aging, and promote the biosynthesis of glycosaminoglycans and collagen. In the drug delivery sector, numerous recent studies have reported on nanocarriers modified with LBA for the controlled release of anticancer drugs [9,10,11,12,13]. Examples include poly (lactide-co-glycolide; PLGA) nanoparticles with LBA surface functionalization via the amino groups of chitosan for the delivery of the drug etoposide to retinoblastoma cells [14]; porous PLGA nanoparticles conjugated with LBA for the controlled release of silymarin (a flavonoid extract of Silybum marianum) to inhibit the proliferation of human liver cancer cells and treat hepatocellular carcinoma [15]; PLGA nanoparticles coated with chitosan modified with polyethylene glycol (PEG) and LBA, for the targeted release of arsenic trioxide to the liver tumor [13].

Systems based on zein often make use of oleic acid (OA) as a softening/toughening component for films or hot-melt extruded products. Oleic acid is considered an emulsifying agent to improve the film formation characteristics in mixtures with various biocompatible organic compounds [16]. For instance, the addition of amphiphilic plasticizer fatty acids (e.g., palmitic acid and stearic acid) was used to produce protein-lipid sandwich structures in films, leading to a decrease in water absorption characteristics by reducing the extent of direct exposure of zein to water [17]. Similar decreases in water absorption of zein films derived from oleic, linoleic, and lauric acid plasticization were also observed in other studies [18,19].

The work presented here explored the potential of the unprecedented combination of lactobionic acid and zein (a plant protein derived from maize) in mixtures suitable for products, such as films, exhibiting a controlled release of LBA to provide antioxidant and antibacterial protection. Zein is insoluble in water but soluble in aqueous ethanol solutions; it is widely used to produce drug delivery systems because of its biocompatibility, biodegradability, and Generally Recognized as Safe (GRAS) status [20,21,22]. Another novel feature of the work was the incorporation of oleic acid as a means of controlling the properties of the films, which could be particularly beneficial for cosmetic applications. In this study, zein-based films with a multi-phase morphology were produced to enable a rapid release of LBA into an aqueous physiological solution when used either as a single additive or in combination with oleic acid. The latter was not expected to leach out from the films owing to its hydrophobic nature. The anticipated radical scavenging characteristics of the LBA species released from the films were demonstrated alongside the effective inhibition of bacterial growth. Although this work was intended to be a feasibility study, the results were expected to be useful for several specific applications, particularly in skincare products and food packaging.

## 2. Materials and Methods

### 2.1. Preparation of Zein-Based LBA Films

Solutions of maize zein (Sigma Aldrich) were prepared at a concentration of 20% (*w*/*v*) in a 70% *v/v* ethanol solution in deionized water at room temperature [23]. Solutions of LBA (HPLC-grade obtained from Sigma-Aldrich) were prepared at a concentration of 20% (*w*/*v*) in deionized water. The solutions of the two individual components were used to prepare zein-LBA mixtures (ZL) containing 25% LBA and 75% zein, both corresponding to weight percent solid content. The zein-oleic acid LBA (ZOL) system was prepared with the following solution volumetric ratios: 26.7% oleic acid (OA), 40.0% zein, and 33.3% LBA, using the same procedure adding the oleic acid component from a solution in ethanol (80 *v*/*v*%). The zein solution (without LBA or OA) was used to prepare control films for release, antioxidant, and antimicrobial assays.

The resulting mixtures were poured into 50 mm diameter polystyrene Petri dishes, left to dry overnight under the fume cupboard, and treated at 60 °C for 24 h in a hot air circulating oven. This procedure enabled the production of zein-based LBA-containing films.

### 2.2. Differential Scanning Calorimetry

The differential scanning calorimetry (DSC) instrument used was a DSC Q20 V24.11 Build 124 equipped with the Universal V4.5A TA Instrument software for data analysis. Heat-cool-heat scans at 10 °C/minute between −80 °C and 180 °C were carried out to establish the main thermal transitions of zein-based LBA-containing films with the view of determining the degree of miscibility of the components in the mixtures. The first heating cycle was used primarily to eliminate the effects of water possibly entrapped in the system. A calibration procedure, according to ASTM E967 and E968 test methods (PN 915060.901), was adopted using indium reference material.

### 2.3. Dynamic Mechanical Analysis

Specimens of 25.0 × 8.0 × 0.1 mm^3^ of zein films (control) and 25.0 × 8.0 × 0.2 mm^3^ of zein-LBA films were tested in N_2_ atmosphere using a dynamic mechanical analysis (DMA) apparatus Rheometric Scientific DMTA V (Perkin Elmer, Waltham, MA, USA) working at 1 Hz and in a range of temperatures between −120 °C and 150 °C. The upper temperature used was below the *T*_g_ of zein but above the *T*_g_ of LBA, as confirmatory data for a possible degree of miscibility of the two components. A preliminary strain sweep test was performed to determine the limit for a linear viscoelastic behavior of the samples.

### 2.4. Scanning Electron Microscopy

Scanning electron microscopy (SEM) micrographs of the zein-based LBA-containing films were taken for both the surface and the cross-section of the samples before and after the release tests. Samples were fractured after cooling in liquid nitrogen, fixed on aluminum stubs by graphite paint, and gold-sputtered in a vacuum using a coater (Quorum 150R ES Quorum Technologies Ltd., East Sussex, UK). The SEM system used was XL20 (Philips, FEI, Hillsboro, OR, USA), equipped with a secondary electron detector and operating at 10 kV voltage.

### 2.5. Release of LBA from Zein-Based Films

Films of zein-LBA and zein-LBA-OA (50 mm diameter; 0.3 mm in thickness) were weighed and then immersed in 120 mL of a physiological solution (9 g/L of NaCl in distilled water) at 60 °C (to accelerate the LBA release) and stirred at 500 rpm. This temperature was well below the *T*_g_ of the zein matrix and, therefore, was not expected to alter the mechanism for the release of LBA from that expected at the ambient temperature. At the same time, the pH of the physiological solution was left at its natural neutral state to avoid possible complications in the release mechanism owing to the acidic nature of the additives in the zein-based films. The diffusion of LBA was monitored by measuring the pH of the solution using a pH meter (Eutech™ pH 700 Meter) concurrently with measurements of the LBA concentration by high-performance liquid chromatography (HPLC), taking aliquots (1 mL) from the test solution at different time intervals. The samples were previously filtered through a cellulose acetate membrane (0.22 µm–25 mm) before HPLC analysis. HPLC analyses were carried out in a liquid chromatography system (Agilent 1260 Infinity) equipped with an Agilent Hi-Plex Ligand Exchange Columns (H^+^) 300 × 7.8 mm, coupled to a refractive index detector. The mobile phase consisted of a 5 mM H_2_SO_4_ solution at a flow rate of 0.6 mL/min. Released LBA was quantified according to HPLC-grade external analytical standard LBA obtained from Sigma-Aldrich (Milan, Italy). Data acquisition and analysis were performed with ChemStation software (Agilent). All results were presented as the average of two independent experiments.

### 2.6. Antioxidant Capability of Zein-LBA Films

The antioxidant activity of the LBA released from the zein-LBA films was evaluated by the standard method of 2,2-diphenylpicrylhydrazyl (DPPH), according to the literature [24]. The system containing oleic acid was not examined as it was established that OA does not leach out to any significant extent in the extraction tests and since the quick release of LBA does not support the use of such films in the continuous prolonged release of the drug. The 1 mM (in ethanol) DPPH stock solution was used for the dilution of the sample to reach a DPPH final concentration of 0.1 mM. Starting from a concentration of LBA as the final amount obtained in the extraction/diffusion assay (1680 mg/L), the concentration of the extracted LBA was reduced in steps over a wide range. The same solution and DPPH ratio were used for zein films (without LBA) as a negative control after the same period of extraction, while the 0.1 mM DPPH solution was used as blank. After the addition of DPPH to each sample (final reaction volume of 1 mL), the cuvette was kept at room temperature in a dark chamber for 30 min to avoid a possible radical reaction caused by the light. The absorbance at λ = 517 nm was read (using a double ray method), and the data were plotted to determine the Q factor, using the equation:

(1) $$Q\;factor=\left(\frac{A_{0}-\;A_{C}}{A_{0}}\right)\times 100$$

where *A*_0_ is the absorbance of the control (zein film) and *A_c_* is the absorbance of the sample analyzed (zein-LBA film). The *Q* factor was then used to determine the EC_50_, defined as the LBA concentration needed to decrease the initial absorbance of the sample by 50% of the original value.

### 2.7. Antimicrobial Activity of Released LBA

To evaluate the antimicrobial activity of the zein-LBA films, Gram-negative *Escherichia coli* DH5-alpha and Gram-positive *Staphylococcus epidermidis* ATCC14990 were used as indicator strains. As for the studies on the antioxidant activity, the system containing oleic acid was not examined as it was established that OA does not leach out to any significant extent in the extraction tests. In addition, the quick release of LBA from zein-LBA-OA films limited the application of these films for the prolonged release of LBA. The bacterial strains were cultured (150 rpm, 16 h, 37 °C) in 100 mL flasks containing 20 mL of diluted Luria–Bertani broth (dLB: 1/5 strength LB). The cells in the exponential phase (precultures) were used as inoculum (5% *v*/*v*). Agar well-diffusion assay was used to evaluate the antimicrobial activity of zein-LBA films. Briefly, the agar plate surface was inoculated by pouring 5 mL of soft dLB agar (7.5 g/L) containing 10^5^–10^6^ CFU/mL of indicator strain. Then, zein-LBA and zein films (negative control), previously sterilized by exposure to ultraviolet (UV) light, were laid on the surface of the agar. After 24 h incubation at 37 °C, the presence of a clear zone around the zein-LBA films was considered as evidence of the antimicrobial activity of LBA. In addition, precultures were inoculated in a flask containing 20 mL of dLB broth and zein-LBA films (~70 mg weight; 0.3 mm thickness; LBA content ~0.70 g/L) previously sterilized by exposure to UV light. The cultures were incubated on an orbital shaker at 150 rpm and 37 °C for up to 29 h. Samples were aseptically withdrawn after 0, 2, 4, 6, 24, and 29 h incubation after the addition of the sterilized film for growth monitoring. Planktonic bacterial growth was monitored by determining the number of colony-forming units (CFU) per mL using the drop plate method [25], and growth curves were established by plotting the log_10_ CFU/mL as a function of time. As controls, dLB both without and with zein films inoculated with 5% preculture or uninoculated (abiotic) were used. The inhibitory activity was expressed as inhibition percentage calculated according to the following equation:

(2) $$\mathrm{Inhibition}\;\left[\%\right]=\frac{\mathrm{CFU}/{\mathrm{mL}}_{\mathrm{control}\left(\mathrm{zein}\right)}-\mathrm{CFU}/{\mathrm{mL}}_{\mathrm{sample}\;\left(\mathrm{zein}-\mathrm{LBA}\right)}}{\mathrm{CFU}/{\mathrm{mL}}_{\mathrm{control}\left(\mathrm{zein}\right)}}\times 100$$

### 2.8. Statistical Analyses

Results of log_10_ CFU/mL of *E. coli* and *S. epidermidis,* grown in the presence of zein-LBA films or under standard conditions (zein films without LBA), were statistically evaluated using multiple unpaired *t*-tests. Statistically significant results were depicted by *p*-values < 0.05. All analyses were performed using Graph-Pad Prism version 8.0.0 for Windows (GraphPad Software, San Diego, CA, USA).

## 3. Results and Discussion

### 3.1. Morphology and Physical State of Mixtures

**Figure 1:**
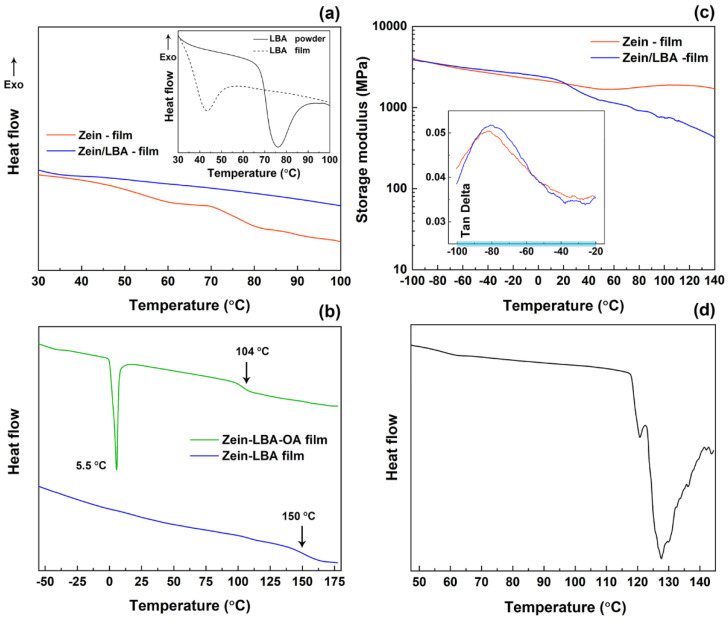
Differential scanning calorimetry (DSC) thermal data and dynamic mechanical analysis (DMA) spectra. (**a**) Thermograms of zein and zein-LBA (lactobionic acid) films and β relaxations thermograms of LBA with different thermal history (inset). (**b**) DSC thermograms for zein-LBA and zein-LBA-OA (oleic acid) films. (**c**) Variation of storage modulus with temperature, with tan δ variation in the β relaxation regions. (**d**) Thermogram for LBA for the entire spectrum from β relations to the glass transition.

The complexity of the thermal transitions exhibited by LBA has been discussed in our previous work [6], which established that the behavior is very sensitive to the thermal history of the sample. It was shown that the main glass transition of LBA started at around 122 °C, while another transition (*T*_β_), associated with β relaxations, occurred at around 75 °C, which was very sensitive to the moisture present in the sample. The thermogram in the inset of Figure 1a shows that the *T*_β_ peak temperature decreased to a value around 40 °C for a film cast from water solution and dried at ambient temperature. The thermograms for films produced from the zein-LBA (ZL) mixture of this study (Figure 1a) was completely devoid of any thermal transition features except for a small depression at around 35 °C, which could be attributed to the presence of segregated LBA domains containing small quantities of residual water. The undulated nature of the thermogram for the zein film, on the other hand, could be regarded as a peculiar feature of the system.

A comparison of DSC thermograms for the zein-LBA (ZL) and the zein-LBA-OA (ZOL) mixtures is shown in Figure 1b. This displayed a smooth trace for the ZL system with a clear transition at around 150 °C, in correspondence with typical values found for the *T*_g_ of zein [26]. A very small transition seemed to be evident at around 120 °C, possibly associated with the LBA component present as a separate phase. The thermogram for the ZOL system, on the other hand, showed a clear glass transition at 104 °C, which was attributed to the plasticization of zein and a sharp melting transition (Tm) at 5.5 °C with a melting enthalpy equal to 10.8 J/g. This peak was associated with the presence of oleic acid (OA) as a segregated discrete phase. In this context, it should be noted that the melting and fusion behavior of pure oleic acid is confounded by its polymorphic nature, consisting of two reversible crystallographic structure (γ and α) with melting points equal to −48 °C and 5 °C, respectively. However, these values can change considerably in mixtures due to the formation of eutectics [27]. Nonetheless, the Tm value in Figure 1b coincided with that reported for the α form of pure oleic acid. It was instructive to observe that the recorded ΔH_m_ value of 10.8 J/g for the crystallized OA in the DSC run increased to approximately 18 J/g when normalized with respect to the total amount of OA in the mixture (ΔH_m_ for pure OA = 75.5 J/g) [27]. This suggested that about 50% of the OA present in the mixture crystallized out.

The variation of both storage modulus and tan δ with the temperature obtained from the DMA tests for zein and zein-LBA films is shown in Figure 1c. Note that it was not possible to perform DMA tests on the zein-LBA-OA mixture due to the very soft and fragile nature of the films. The trendline for the storage modulus of the zein film displayed the start of the glass transition at around 140 °C and a small trough at around 50 °C, resulting from the unconstrained loss of free water, which could be associated with the grainy structure of zein. This behavior was consistent with the data reported by Singh et al. [28]. Although this effect was less pronounced in the present work, the data of these authors showed a trough in the traces of both storage and loss modulus at around 50 °C followed by a peak at higher temperatures.

Other data presented in the work of Singh et al. indicates that the depression of this peak can be related to the enhanced water holding capacity of the zein films when small quantities of iodine are incorporated as a complexing agent [28]. Accordingly, the monotonic decrease in the storage modulus from a temperature above 20 °C for the zein-LBA films of this present study may be attributed to moisture strongly bound within the LBA domains or at the zein/LBA interface. This resulted in a depression in the rate of water release from the specimen during the test, causing a plasticization effect, which manifested as a decrease in the modulus. To demonstrate the high water-holding capacity of LBA, an ad hoc experiment was conducted on a mixture of LBA powder with 5 wt% water that was subjected to a DSC analysis after one day, using the same experimental conditions of the zein-LBA films. The corresponding thermogram in Figure 1d (bottom right) showed the lack of a significant effect of small amounts of absorbed water on the *T*_g_ of LBA (~120 °C), which was explosively released upon reaching the glass transition temperature. Similar behavioral peculiarities of molecular glasses, arising from a strong intermolecular attraction with water, have been widely reported in the literature [29] (the interactions of LBA with water are also discussed later in relation to the extraction tests). Note that the exotherm in the LBA thermogram above the glass transition was associated with thermal decomposition events.

A significant feature of the mechanical spectra of the films of this study was the β transition of zein, which was manifested by the two traces of the tan δ variation with the temperature in the inset of Figure 1b. The two spectra were almost identical in displaying a distinct sharp peak at around −80 °C, which was indicative of a lack of miscibility between zein and LBA, while the slight narrowing of the peak for the zein-LBA films, if significant, might be attributed to the presence of small quantities of water at the interface. It should be noted that miscibility would induce a flattening and displacement of this peak along the temperature axis [29,30].

### 3.2. Release of LBA from the Zein-Based Films

**Figure 2:**
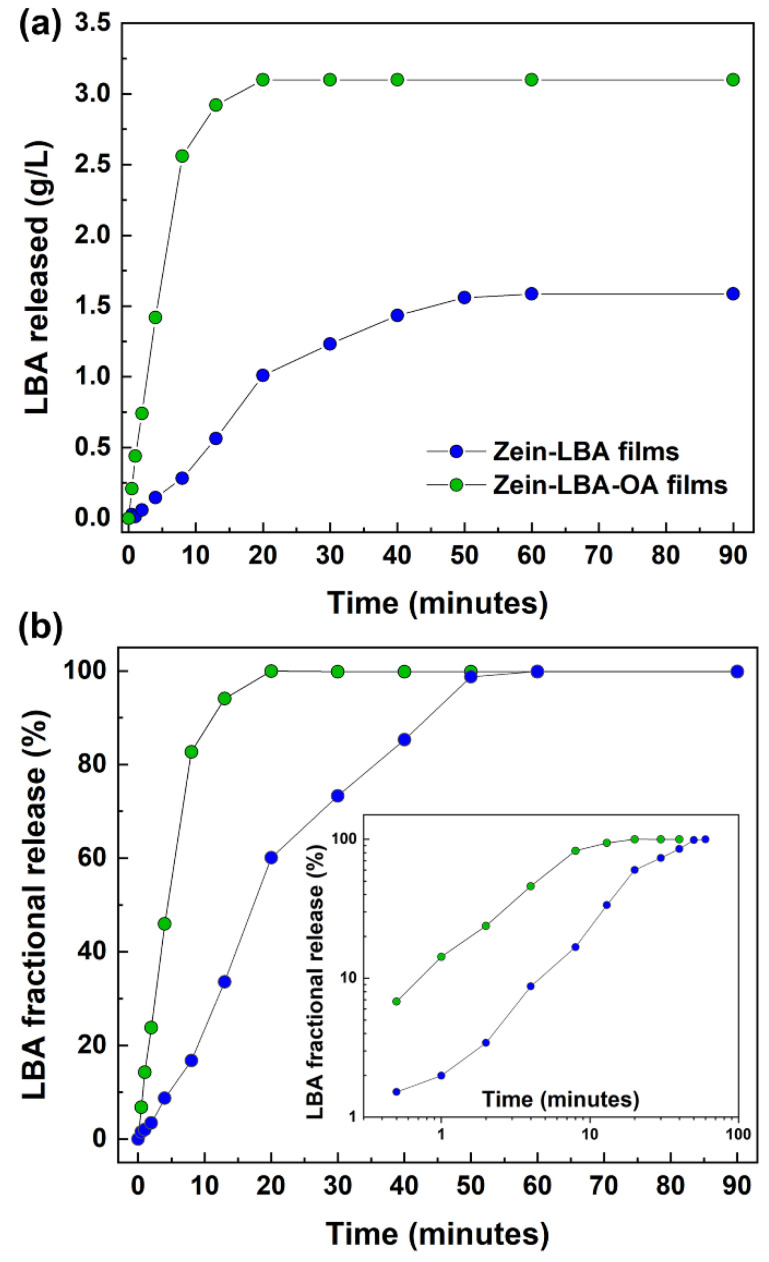
Evolution of LBA release in the physiological solution from zein-based films. (**a**) As recorded; (**b**) Normalized values with respect to the total release for the same systems, with corresponding data in the inset in the form of a log-log plot (Peppas Power law diffusion [31]).

LBA release experiments were performed over a period of 90 min in all cases. A rapid accumulation of extracted LBA in the physiological solution was recorded for both zein-LBA and zein-LBA-OA films through HPLC measurements carried out at different time intervals (Figure 2a). The final concentration of LBA for the zein-LBA film was 1.68 g/L, corresponding to a 23.76 wt% total extraction from the film (95.0 wt%), while for the zein-LBA-OA film, the terminal LBA concentration was 3.10 g/L (93.7 wt%). Considering that the purity of the LBA used is stated to be 97%, total extraction can be considered to have occurred in both systems. An initial burst release of LBA was observed from both films, with 1/3 of LBA released in 3.5 min for the zein-LBA-OA film and 12 min for zein-LBA, reaching total extraction in 20 and 50 min.

The normalized values with respect to the terminal LBA concentration are shown in Figure 2b as the fractional concentration of LBA for a quantitative comparison regarding the relative rates of extraction. These highlighted the almost total release of LBA in both cases and a much faster release rate of the zein-LBA-OA system. The log-log plots in the inset indicated that the data followed approximately the Peppas Power law diffusion behavior from the start, slowing down in the stages due to the large reduction in concentration gradient [31]. According to the power law expression for the ratio of the extracted amount at time *t*, (*Q_t_*), to the final amount (*Q*∞), i.e., *Q_t_/Q*∞ *= K t^n^*, of zein-LBA-OA films displayed a larger exponential index *n* than the corresponding zein-LBA system, which indicated that the presence of oleic acid was changed to the extraction mechanism [32].

**Figure 3:**
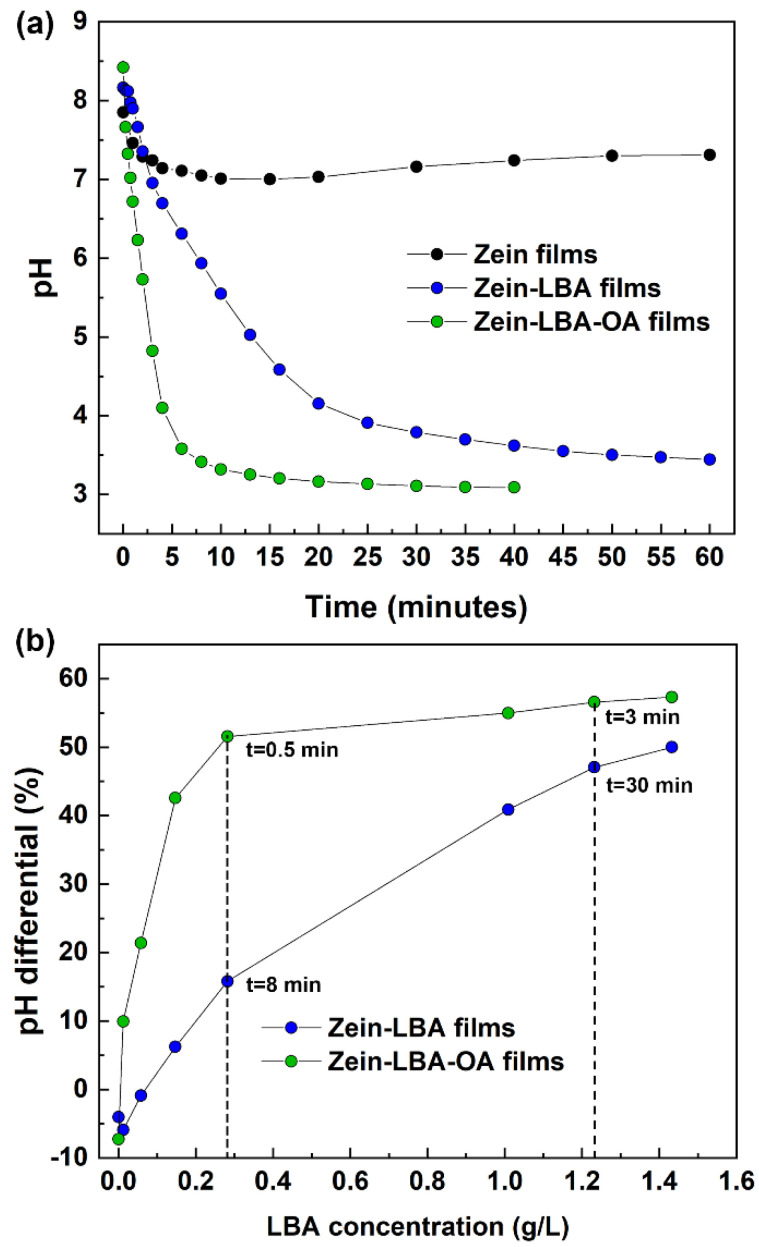
(**a**) Variation of pH of the extraction solution with time for the three films examined. (**b**) The plot of pH differential against LBA concentration in the extraction time at two different stages of the extraction process for both zein-LBA and zein-LBA-OA systems.

A progressive decrease in the pH of the physiological solution was registered during the same extraction period for both zein-LBA and zein-LBA-OA films, reaching a corresponding plateau after 60 and 20 min, respectively (Figure 3a). In the same Figure, a plot of the values recorded for the neat zein film (without LBA) as a control to provide a baseline for the change in pH resulting from the release of the two added acids in the mixture is included. In this respect, it should be noted that the diffusion assay performed on neat zein films (without LBA), under the same conditions, displayed a small drop in pH from 7.85 to 7.31, reaching a “quasi-plateau” after 20 min extraction. The plots in Figure 3a display a substantial reduction in pH within the first 5 min in all cases until neutral conditions at pH = 7 were reached. This behavior is associated with the adsorption of OH^-^ ions from the physiological solution onto the surface of the films, which was consistent with data reported in the literature quoting the isoelectric point of zein at pH = 6.8 and acquiring net positive charges at a higher pH [33]. In this respect, it is useful to note the related observations by the same authors regarding the peculiar changes in the zeta potential for zein nanoparticles mixed with 6,7-dihydroxycoumarin at different pH values when immersed in water containing various amounts of NaCl. Very large variations in zeta potential values were observed at pH = 8, with negative values at pH > 5 and positive values at pH < 5.

Figure 3b shows the plots of the % pH differential between the two formulated films and zein, as % reduction relative to zein, Δ(pH), in the function of the LBA concentration in the extraction solution for the two films at different stages:

(3) $$\%\Delta (\mathrm{pH}{)}_{\mathrm{ZL}}=[\mathrm{pH}\;\left(\mathrm{zein}\right)-\;\mathrm{pH}\left(\mathrm{ZL}\right)/\mathrm{pH}\left(\mathrm{zein}\right)]\;\times \;100$$

(4) $$\%\Delta (\mathrm{pH}{)}_{\mathrm{ZOL}}=[\mathrm{pH}\;\left(\mathrm{zein}\right)-\;\mathrm{pH}\left(\mathrm{ZOL}\right)/\mathrm{pH}\left(\mathrm{zein}\right)]\;\times \;100$$

**Figure 4:**
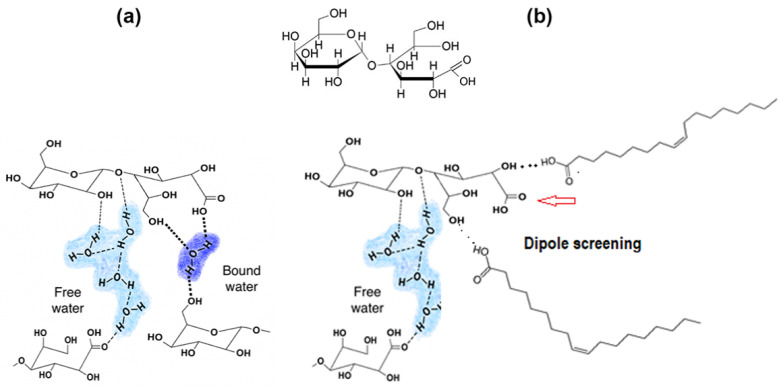
Structure of lactobionic acid [2] and molecular interactions with species in zein-based films; (**a**) weak and strong H-bonds with water; (**b**) weak polar interactions with oleic acid.

The curves in this figure show that for the same concentration of LBA in the extraction solution the pH reduction was always lower for the system containing oleic acid (zein-LBA-OA). While this was to be expected from the higher content of LBA of the latter films, the peculiar large difference at intermediate extraction times suggested that the extraction of small amounts of oleic acid might form “complexes” through the interactions with lactobionic acid. These reduced the intensity of the intermolecular association, which brought about a concomitant increase in the degree of ionization of the COOH groups (see also the later discussion about Figure 4b). Towards the terminal stages of the extraction, resulting in an accumulation of LBA, the relative OA concentration in the solution became progressively lower and, therefore, it was expected that this would reduce the effect on the depression of the ionization of the COOH groups. The plot in the inset of Figure 1 also revealed the anomaly at the start of the extraction process, where the pH differential assumed negative values due to the presence of acids in the mixture thereby providing a larger number of H^+^ available for adsorption on the surface of the zein domains.

It should be noted that although the sodium chloride ions in the physiological solution can be involved in the molecular associations of oleic acid to form Na oleate species, these constitute only a small fraction of the total OA at pH less than 7 [34].

In Figure 4, the molecular interactions occurring within the LBA domains of the mixtures are depicted. The strong H-bonds between COOH groups and water in LBA could form intermolecular associations (bound water), which m slow down the motions within the hydrated LBA species involved in the extraction from the film. It would be difficult to obtain confirmation of the interaction by infrared spectroscopy (FTIR) as the spectra would be dominated by the extensive overlap of absorption bands due to the communality of the groups in the two components.

The water dissolution of LBA, on the other hand, was expected to take place through the formation of weak attractions between hydroxyl groups giving rise to the accumulation of water within the LBA domains (free water), as indicated in Figure 4a.

The COOH groups of free oleic acid species can be expected to exhibit strong polar interactions with the multitude of OH groups of lactobionic acid, thereby forming a shield for the acid groups of LBA against the access of the required amount of water required for ionization to produce H^+^ protons, as shown in Figure 4b.

**Figure 5:**
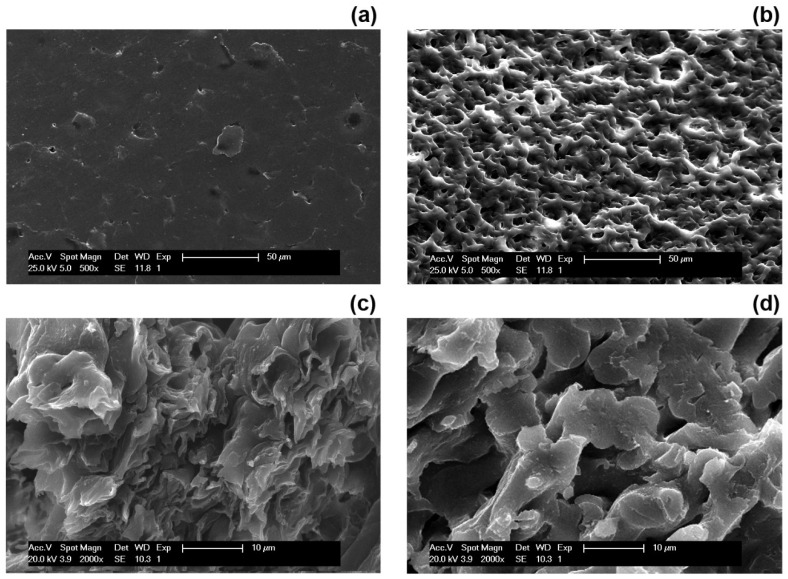
Scanning electron microscopy (SEM) images of surfaces and cross-sections of zein-LBA films before and after LBA release: surface (**a**) before extraction, (**b**) after extraction; cross-section (**c**) before extraction, (**d**) after extraction. Scale bars: 50 µm for (**a**,**b**); 10 µm for (**c**,**d**).

The morphology of the zein-based films examined by SEM is shown in Figure 5. Here, micrographs of the zein-LBA films, both as produced (Figure 5a,c) and after the total extraction of LBA (Figure 5b,d), were reported. The SEM micrographs taken on the surface of the zein-LBA films before extraction revealed the presence of dispersed domains, while the fractured surface showed the presence of a mixture of rugged grainy domains with a sharp interface normally associated with a lack of molecular miscibility of the components of the film. In contrast, the surface cratering feature and porosity of the films increased after the complete release of LBA, revealing the presence of interconnected pores both on the surface and through cross-section, which indicated that the two components (zein and LBA) in the film were present as co-continuous domains. Bearing in mind that the fraction of LBA present in the mixture was only ¼, the bi-continuous morphological featured of the two phases would not take place if the two components were not interactive and the precipitating LBA moieties possessed a strong thermodynamic drive to grow into particles, as in crystallization processes.

These observations suggested that in the formation of the film, through evaporation of the solvent (ethanol/water mixture), zein separated out first due to the more rapid evaporation of ethanol. This would produce a very viscous medium (swollen hydrogel), which slowed down the motions of the dissolved LBA molecules, “freezing” at the same time the physical state of the phase separating species (spinodal decomposition) and developing into fine co-continuous water-swollen diffused domains. The formation of this type of morphology was assisted by both the lack of a crystallization drive for the LBA (as mentioned earlier) and the high interface free energy between the two phases, which assisted the coarsening of the 3D interpenetrating LBA domains to reach values in the region of 5–10 μm during the evaporation of water. The coarse nature of the co-continuous domains of these zein-based systems alongside the presence of sharp interfaces was mostly responsible for the rapid release of LBA from the films. The coarse microstructure of the co-continuous domains of the zein-based systems and the presence of sharp interfaces were mostly responsible for the rapid release of LBA from the films. This was in contrast with the polymer-based organic-inorganic hybrids, which were designed to impart a very slow release of additives such as antioxidants [35] and corrosion inhibitors [36]. In these latter systems, phase co-continuity could be obtained at low contents of the encapsulating pseudo-inorganic component (hybrid) by inducing chemical reactions at the interface during phase separation, which prevented the coarsening of the 3D domains.

**Figure 6:**
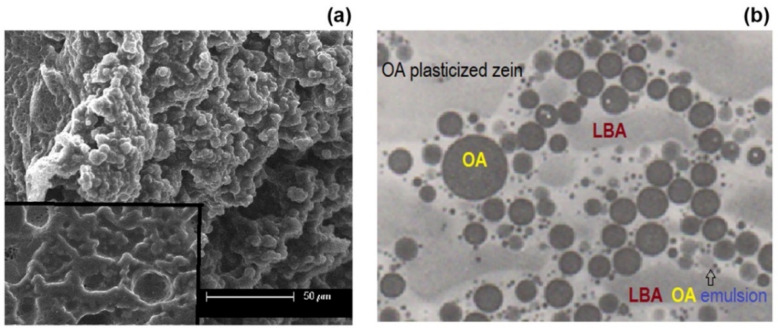
Morphological features of zein-LBA-OA films: (**a**) SEM image of a fractured sample before extraction, with inset for a surface image after extraction; scale bar: 50 μm. (**b**) Schematic illustration, which is based on TEM images of morphologically complex polymer alloys, such as blends of polycarbonate (PC) and ABS (acrylonitrile-styrene-butadiene terpolymer), containing encapsulated rubber particles within a continuous matrix of miscible PC/SAN [37].

Figure 6a shows a micrograph of the cross-section of the zein-LBA-OA film before extraction with an inset consisting of a micrograph taken on the surface of the film after extraction. This displayed a different morphology than the binary component zein-LBA film, which was substantially particulate within a continuous matrix. The presence of particles was still visible after extraction, which could be identified as oleic acid domains intermingled with LBA particles in the pristine film. There were also numerous fine particles, which appeared to have been attached to much larger particles. It was envisaged that these consisted of emulsified particles of LBA through surface associations with oleic acid, as indicated in Figure 6b.

### 3.3. Antioxidant Activity of Zein-LBA Films

**Figure 7:**
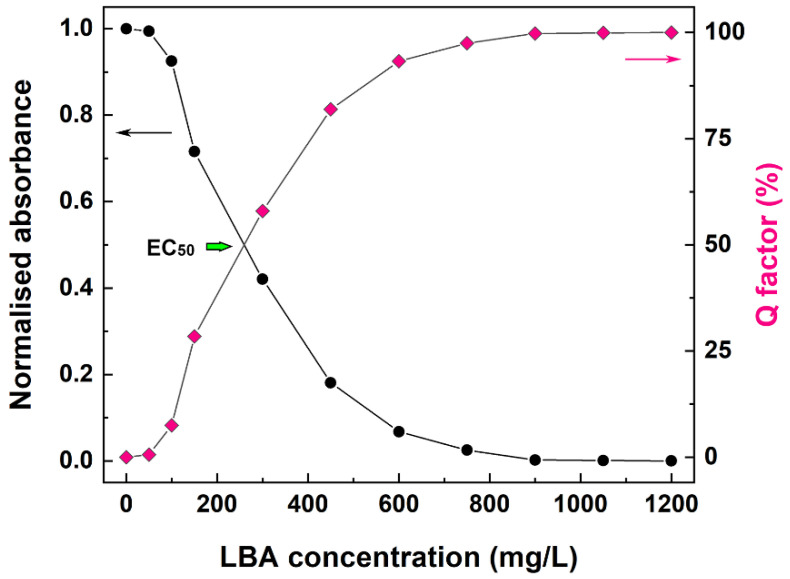
Results of the 2,2-diphenylpicrylhydrazyl (DPPH) antioxidant assay. Normalized absorbance at 517 nm (black circles, *y*-axis on the left-hand side) and *Q* factor values (magenta diamonds, *y*-axis on the right-hand side) versus LBA concentration released from the zein-LBA films. The green arrow indicates the value of EC_50_.

The antioxidant characteristics of LBA released from the zein films were evaluated using the DPPH antioxidant activity assay, with zein films (without LBA) as control. The experimental data are plotted in Figure 7 as normalized absorbance (*λ* = 517 nm) and calculated *Q* factor as a function of the LBA concentration in the assay solution. The *Q* factor was used to estimate a value of 256 mg/L for EC_50_ as a parameter indicative of strong antioxidant activity of the zein-LBA films, notwithstanding the difficulties arising from the dependence of the EC_50_ value on the concentration and nature of the experimental conditions [38].

In this respect, it should be noted that Tasic-Kostov et al. have reported an EC_50_ value of 53.63 mg/mL for LBA in an emulsion of 6 wt% concentration in C16/18 APG-mixed emulsifier–cetearyl glucoside and cetearyl alcohol [3]. EC_50_ values up to 8.6 mg/mL were instead measured for low molecular weight chitosan-LBA derivatives [39]. For the zein-LBA films of the present study, a notable difference was the use of a physiological solution (9 g/L of NaCl) as a medium for testing the radical scavenging characteristics of LBA. Considering that no antioxidant effect was observed for zein films (without LBA), the results of this assay confirmed that the recorded DPPH radical scavenging activity was entirely due to the LBA released from the films.

### 3.4. Antimicrobial Activity of Zein-LBA Films against E. coli and S. epidermidis

**Figure 8:**
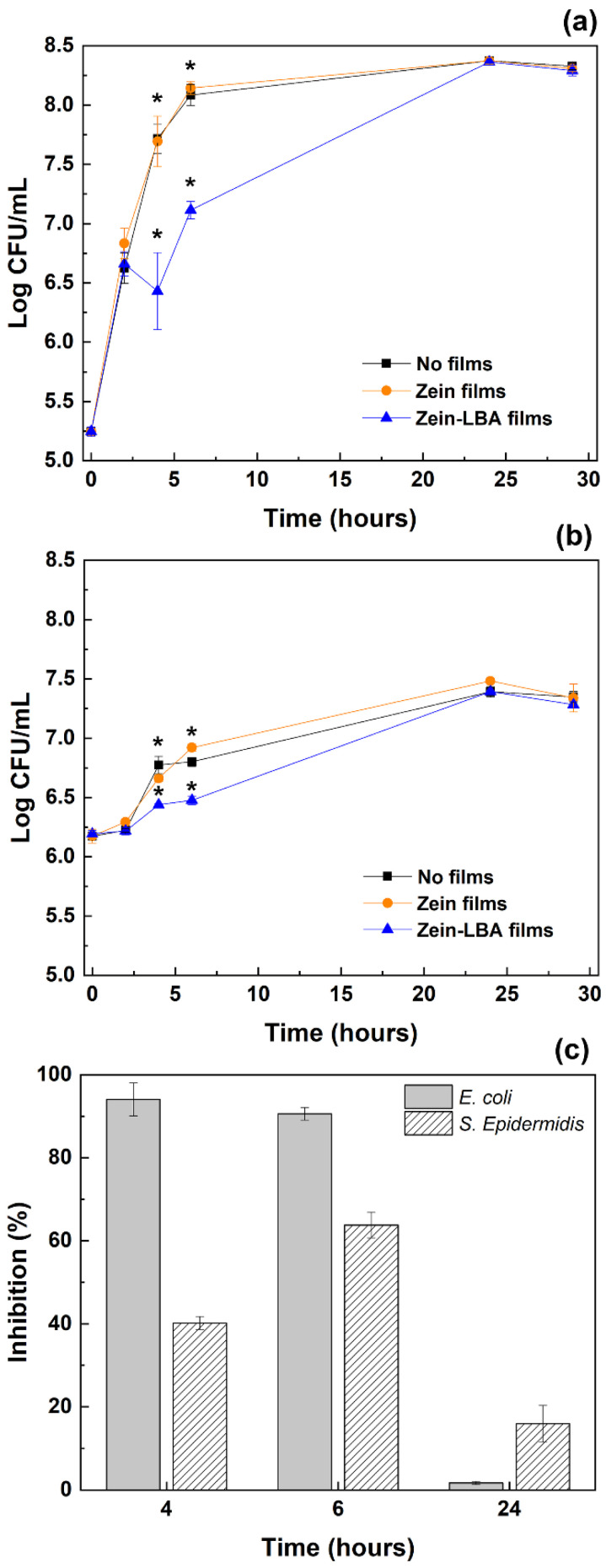
Effect of zein and zein-LBA films on the growth of (**a**) *E. coli* and (**b**) *S. epidermidis* at different time points. The dLB medium was used as control (“no films”). Data are expressed as means ± standard deviations of log_10_ CFU/mL during microbial growth in dLB without films and with zein and zein-LBA films. Values with “*” are significantly different at *p* < 0.05. (**c**) Inhibition percentages for zein-LBA films for the two bacterial strains at different time points (4, 6, and 24 h).

The antimicrobial activity of zein and zein-LBA films (LBA amount ~0.70 g/L) was tested against *E. coli* and *S. epidermidis* as model microorganisms (Figure 8a,b). It was deduced, therefore, that zein films had no effect on both bacterial strains due to the average cell count (log_10_ CFU/mL) being the same as that of the control group (dLB medium). These results were in good agreement with previous works reporting that zein films were not effective to inhibit the growth of different Gram-positive and Gram-negative bacterial species [39]. Conversely, in the present study, an evident growth variation was observed for *E. coli* and *S. epidermidis* when the strains were grown in the presence of zein-LBA films, both in agar diffusion assay and liquid medium (Figure S1 and Figure 8). Specifically, in a liquid medium, a predominantly inhibitory effect was observed in the interval between 4 and 6 h, with reductions of 1.26 log_10_ CFU/mL and 0.44 log_10_ CFU/mL for *E. coli* and *S. epidermidis*, respectively. The inhibitory effect was more pronounced for *E. coli* compared to *S. epidermidis*. For *E. coli*, inhibition percentages of 94 ± 4% and 91 ± 2% were recorded after 4 and 6 h of incubation, respectively, while the highest inhibition percentage (64 ± 3%) was recorded for *S. epidermidis* after 6 h of incubation (Figure 8c).

The different inhibition percentages observed for the two indicator strains could be explained by the differences in the cell wall structure of the microorganisms. These results suggested that the complete release of LBA led to bacterial growth inhibition, as confirmed by HPLC analyses performed on the growth medium (Figure S2). At 24 h of incubation, both strains overcame the antimicrobial effect of LBA, and log_10_ CFU/mL values equal to the controls (dLB and zein film) were reached. This was indicative of a bacteriostatic effect of the released LBA at the concentration used in the antimicrobial assay (~0.70 g/L). Previous studies have reported that LBA concentrations higher than 0.70 g/L exert bactericidal activity when used against Gram-positive and Gram-negative bacteria [40,41,42,43].

## 4. Conclusions

In this study, it is shown that zein-based films, encapsulating interconnected domains of LBA hold potential as drug delivery devices, offering the possibility of accelerating the release of LBA in a physiological solution through the addition of OA as an auxiliary component. From the reported observations it transpires that the zein-LBA-OA is a well-engineered system for the fast release of LBA in an aqueous medium.

DCS and DMA tests performed on the zein-LBA films revealed the total lack of miscibility between zein and LBA, resulting in the formation of co-continuous domains of zein and LBA, as highlighted by SEM imaging. These morphological features were responsible for the burst release characteristics of the system, which was synergized by the incorporation of OA in the precursor mixture used for the preparation of the films.

The released LBA exhibited a potent antioxidant activity and exerted an antimicrobial (bacteriostatic) effect against Gram-positive and Gram-negative bacteria.

It was envisaged that a variety of drug delivery systems (films, hollow microspheres, nanoparticle encapsulations, nanofibers) could be developed and finetuned by combining LBA with other antimicrobial and/or antioxidant biomolecules.

The combination with oleic acid could be particularly useful for cosmetic products and edible food packaging, where the plasticization effect on the zein matrix would constitute an additional benefit as a processing aid for hot-melt extrusion.

## Data Availability

The data presented in this study are available on request from the corresponding author.

## Supplementary Materials

The following are available online at https://www.mdpi.com/article/10.3390/polym13111826/s1, Figure S1: Agar-well diffusion assay for evaluation of antimicrobial activity of ZL and Zein films against *E. coli* (A: ZL film; C: Zein film) and *S. epidermidis* (B: ZL film; D: Zein film), Figure S2: Antimicrobial activity assay: HPLC analysis and pH measurements performed on the growth medium for (a) *E. coli* and (b) *S. epidermidis.*

## References

[1] De Giorgi S., Raddadi N., Fabbri A., Toschi T.G., Fava F. (2018). Potential use of ricotta cheese whey for the production of lactobionic acid by *Pseudomonas taetrolens* strains. New Biotechnol..

[2] Gutiérrez L.-F., Hamoudi S., Belkacemi K. (2012). Lactobionic acid: A high value-added lactose derivative for food and pharmaceutical applications. Int. Dairy J..

[3] Tasic-Kostov M., Pavlovic D., Lukic M., Jaksic I., Arsić I., Savic S. (2012). Lactobionic acid as antioxidant and moisturizing active in alkyl polyglucoside-based topical emulsions: The colloidal structure, stability and efficacy evaluation. Int. J. Cosmet. Sci..

[4] Alonso S., Rendueles M., Diaz M. (2013). Bio-production of lactobionic acid: Current status, applications and future prospects. Biotechnol. Adv..

[5] Cardoso T., Marques C., Dagostin J.L.A., Masson M.L. (2019). Lactobionic Acid as a Potential Food Ingredient: Recent Studies and Applications. J. Food Sci..

[6] Mascia L., Coroli A., Mele E. (2019). Probing the Thermal Transitions of Lactobionic Acid and Effects of Sample History by DSC Analysis. J. Pharm. Sci..

[7] Alonso S. (2018). Exploiting the bioengineering versatility of lactobionic acid in targeted nanosystems and biomaterials. J. Control. Release.

[8] Godse R., Rathod M., De A., Shinde U. (2021). Intravitreal galactose conjugated polymeric nanoparticles of etoposide for retinoblastoma. J. Drug Deliv. Sci. Technol..

[9] Habib S.M., Rehman J.-U., Maharjan R., Kanwal T., Althagafi I.I., Saifullah S., Ullah S., Simjee S.U., Shah M.R. (2020). Synthesis of lactobionic acid based bola-amphiphiles and its application as nano-carrier for curcumin delivery to cancer cell cultures in-vitro. Int. J. Pharm..

[10] Li S., Saw P.E., Lin C., Nie Y., Tao W., Farokhzad O.C., Zhang L., Xu X. (2020). Redox-responsive polyprodrug nanoparticles for targeted siRNA delivery and synergistic liver cancer therapy. Biomaterials.

[11] Upadhyay P., Bhattacharjee M., Bhattacharya S., Ahir M., Adhikary A., Patra P. (2020). Silymarin-Loaded, Lactobionic Acid-Conjugated Porous PLGA Nanoparticles Induce Apoptosis in Liver Cancer Cells. ACS Appl. Bio. Mater..

[12] Abdelmoneem M.A., Elnaggar M.A., Hammady R.S., Kamel S.M., Helmy M.W., Abdulkader M.A., Zaky A., Fang J.-Y., Elkhodairy K.A., Elzoghby A.O. (2019). Dual-Targeted Lactoferrin Shell-Oily Core Nanocapsules for Synergistic Targeted/Herbal Therapy of Hepatocellular Carcinoma. ACS Appl. Mater. Interfaces.

[13] Song X., Wang J., Xu Y., Shao H., Gu J. (2019). Surface-modified PLGA nanoparticles with PEG/LA-chitosan for targeted delivery of arsenic trioxide for liver cancer treatment: Inhibition effects enhanced and side effects reduced. Colloids Surf. B Biointerfaces.

[14] Wang X., He L., Wei B., Yan G., Wang J., Tang R. (2018). Bromelain-immobilized and lactobionic acid-modified chitosan nanoparticles for enhanced drug penetration in tumor tissues. Int. J. Biol. Macromol..

[15] Du H., Liu M., Yu A., Ji J., Zhai G. (2017). Insight into the role of dual-ligand modification in low molecular weight heparin based nanocarrier for targeted delivery of doxorubicin. Int. J. Pharm..

[16] Lai H.-M., Padua G.W., Wei L.S. (1997). Properties and Microstructure of Zein Sheets Plasticized with Palmitic and Stearic Acids. Cereal Chem. J..

[17] Xu H., Chai Y., Zhang G. (2012). Synergistic Effect of Oleic Acid and Glycerol on Zein Film Plasticization. J. Agric. Food Chem..

[18] Paramawati R., Yoshino T., Isobe S. (2001). Properties of Plasticized-Zein Film as Affected by Plasticizer Treatments. Food Sci. Technol. Res..

[19] Paliwal R., Palakurthi S. (2014). Zein in controlled drug delivery and tissue engineering. J. Control. Release.

[20] Raza A., Hayat U., Bilal M., Iqbal H.M., Wang J.-Y. (2020). Zein-based micro- and nano-constructs and biologically therapeutic cues with multi-functionalities for oral drug delivery systems. J. Drug Deliv. Sci. Technol..

[21] Tran P.H., Duan W., Lee B.-J., Tran T.T. (2019). The use of zein in the controlled release of poorly water-soluble drugs. Int. J. Pharm..

[22] Yong Z., Lili C., Feng L., Nianqiu S., Chunlei L., Xianghui Y., Yan C., Wei K. (2016). Design, fabrication and biomedical applications of zein-based nano/micro-carrier systems. Int. J. Pharm..

[23] Mascia L., Zhang W., Gatto F., Scarpellini A., Pompa P.P., Mele E. (2019). In Situ Generation of ZnO Nanoparticles within a Polyethyleneimine Matrix for Antibacterial Zein Fibers. ACS Appl. Polym. Mater..

[24] Esmaeili H., Karami A., Maggi F. (2018). Essential oil composition, total phenolic and flavonoids contents, and antioxidant activity of *Oliveria decumbens* Vent. (Apiaceae) at different phenological stages. J. Clean. Prod..

[25] Herigstad B., Hamilton M., Heersink J. (2001). How to optimize the drop plate method for enumerating bacteria. J. Microbiol. Methods.

[26] De Almeida C.B., Corradini E., Forato L.A., Fujihara R., Filho J.F.L. (2018). Microstructure and thermal and functional properties of biodegradable films produced using zein. Polímeros.

[27] Cedeño F.O., Prieto M.M., Espina A., García J.R. (2001). Measurements of temperature and melting heat of some pure fatty acids and their binary and ternary mixtures by differential scanning calorimetry. Thermochim. Acta.

[28] Singh N., Georget D.M.R., Belton P.S., Barker S.A. (2009). Zein−Iodine Complex Studied by FTIR Spectroscopy and Dielectric and Dynamic Rheometry in Films and Precipitates. J. Agric. Food Chem..

[29] Mascia L., Kouparitsas Y., Nocita D., Bao X. (2020). Antiplasticization of Polymer Materials: Structural Aspects and Effects on Mechanical and Diffusion-Controlled Properties. Polymers.

[30] Ghanbarzadeh B., Oromiehi A.R. (2008). Studies on glass transition temperature of mono and bilayer protein films plasticized by glycerol and olive oil. J. Appl. Polym. Sci..

[31] Ritger P.L., Peppas N.A. (1987). A simple equation for description of solute release I. Fickian and non-fickian release from non-swellable devices in the form of slabs, spheres, cylinders or discs. J. Control. Release.

[32] Bouman J., Belton P., Venema P., Van Der Linden E., De Vries R., Qi S. (2016). Controlled Release from Zein Matrices: Interplay of Drug Hydrophobicity and pH. Pharm. Res..

[33] Podaralla S., Perumal O. (2012). Influence of Formulation Factors on the Preparation of Zein Nanoparticles. AAPS PharmSciTech.

[34] Drzymala J., Mittal K.L. (1989). Chemistry of the Oleic Acid-H_2_O-NaCl System VS pH at 25 °C. Surfactants in Solution.

[35] Mascia L., Capra C., Lavorgna M. (2007). Organic-Inorganic Hybrid Fillers for The Controlled Release of Antioxidants. Macromol. Symp..

[36] Mascia L., Prezzi L., Wilcox G.D., Lavorgna M. (2006). Molybdate doping of networks in epoxy–silica hybrids: Domain structuring and corrosion inhibition. Prog. Org. Coatings.

[37] Herpels J., Mascia L. (1990). Effects of styrene-acrylonitrile/butadiene ratio on the toughness of polycarbonate/ABS blends. Eur. Polym. J..

[38] Amorati R., Valgimigli L. (2015). Advantages and limitations of common testing methods for antioxidants. Free. Radic. Res..

[39] Ruiz-Matute A.I., Cobas A.C., García-Bermejo A.B., Montilla A., Olano A., Corzo N. (2013). Synthesis, characterization and functional properties of galactosylated derivatives of chitosan through amide formation. Food Hydrocoll..

[40] Soliman E.A., Khalil A.A., Deraz S., El-Fawal G., Elrahman S.A. (2012). Synthesis, characterization and antibacterial activity of biodegradable films prepared from Schiff bases of zein. J. Food Sci. Technol..

[41] Kang S., Kong F., Shi X., Han H., Li M., Guan B., Yang M., Cao X., Tao D., Zheng Y. (2020). Antibacterial activity and mechanism of lactobionic acid against Pseudomonas fluorescens and Methicillin-resistant Staphylococcus aureus and its application on whole milk. Food Control..

[42] Kang S., Kong F., Liang X., Li M., Yang N., Cao X., Yang M., Tao D., Yue X., Zheng Y. (2019). Label-Free Quantitative Proteomics Reveals the Multitargeted Antibacterial Mechanisms of Lactobionic Acid against Methicillin-Resistant Staphylococcus aureus (MRSA) using SWATH-MS Technology. J. Agric. Food Chem..

[43] Chen H., Zhong Q. (2017). Lactobionic acid enhances the synergistic effect of nisin and thymol against Listeria monocytogenes Scott A in tryptic soy broth and milk. Int. J. Food Microbiol..

