# Supplementary material for: An In-Vitro Evaluation of the Characteristics of Zein-Based Films for the Release of Lactobionic Acid and the Effects of Oleic Acid
Source: Polymers (Basel). 2021 May 31;13(11):1826. doi: 10.3390/polym13111826 (PMC8198277; doi:10.3390/polym13111826)
Supplement: Supplementary file 1 [file polymers-13-01826-s001.zip › polymers-1222965-supplementary.pdf]

Supporting Material

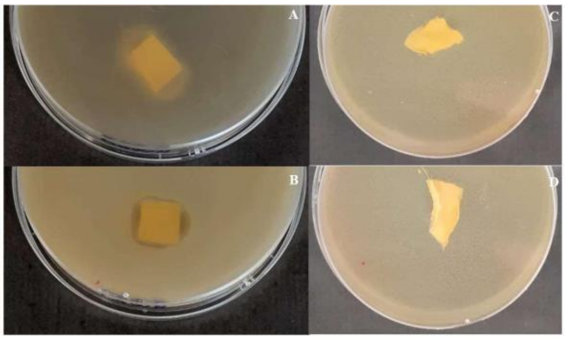

Figure S1: Agar-well diffusion assay for evaluation of antimicrobial activity of ZL and Zein films against *E. coli* (A: ZL film; C: Zein film) and *S. epidermidis* (B: ZL film; D: Zein film).

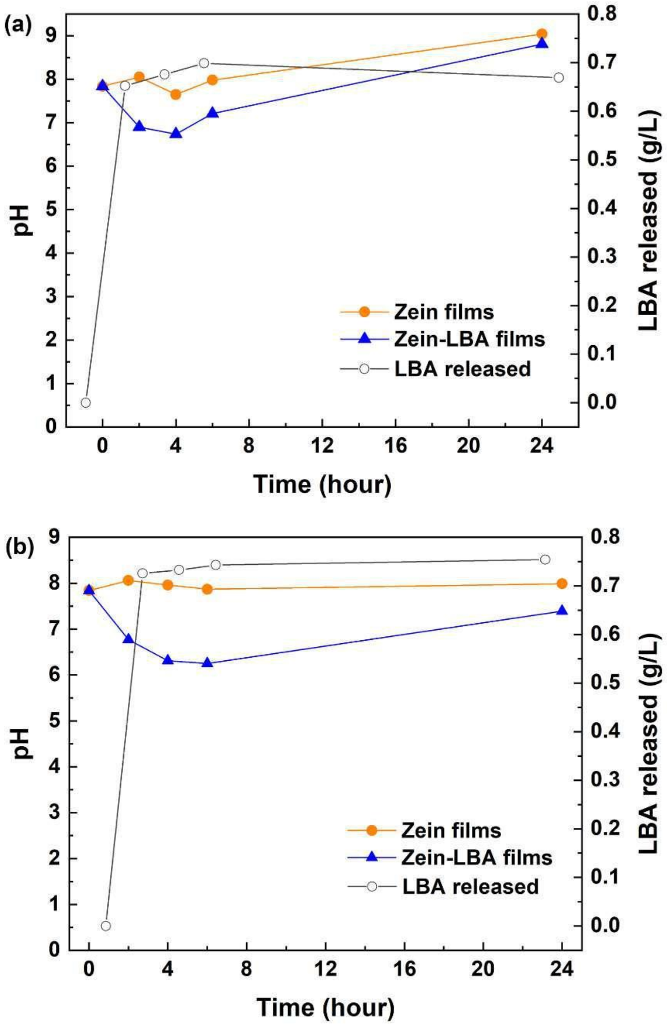

Figure S2: Antimicrobial activity assay: HPLC analysis and pH measurements performed on the growth medium for (a) *E. coli* and (b) *S. epidermidis*.
